# Silver nanoparticles as an antibacterial agent for endodontic infections

**DOI:** 10.1186/1471-2334-12-S1-P60

**Published:** 2012-05-04

**Authors:** W Sofi, M Gowri, M Shruthilaya, Suresh Rayala, Ganesh Venkatraman

**Affiliations:** 1 Department of Human Genetics, Sri Ramachandra University, Porur, Chennai-116, India; 2 Department of Biotechnology, Indian Institute of Technology, Chennai-600 036, India

## Background

Bacteria play an essential role in the initiation, progression, and persistence of dental infections. Therefore endodontic therapy aims to eliminate bacteria from the infected root canal and prevent reinfection.

## Materials and method

Silver nanoparticles have gained more attention owing to their broad spectrum of antibacterial activity and low cost of manufacturing. In the present study, starch coated silver nanoparticle were synthesized and characterized by SEM/EDX and UV/Vis spectroscopy. Starch coated nanosilver was tested for their antibacterial activity against various microorganisms that are commonly found in endodontic failures such as *Enterococcus faecalis*, *Escherichia coli*, *Staphylococcus aureus*, *Streptococcus pneumoniae*, *Acinetobacter baumani*, *Candida albicans*, *Klebsiella pneumonia.* The antibacterial activities were assessed in vitro by 1) Agar diffusion test (ADT) 2) MIC by spectroscopic method 3) efficacy assessment using dentinal tubule model at depths of 200 μm and 400 μm in extracted single rooted teeth.

## Results

The results indicated that the synthesized starch coated nanosilver showed good bactericidal effect against a wide range of organisms. The efficacy study using human tooth model shows that there was a significant reduction in the adherence of *Enterococcus faecalis* to nanoparticulates-treated dentin.

## Conclusion

These experimental results highlighted the potential advantage of silver suspension in root canal disinfection and thereby reduces bacterial invasion into dentin. Hence this eco friendly starch coated silver nanoparticle could be developed as a potent antibacterial agent against a wide range of microorganisms to control and prevent the spreading and persistence of endodontic infections.

